# The Impact of Positive Peritoneal Cytology on the Survival of Endometrial Cancer Patients

**DOI:** 10.3390/diagnostics14192160

**Published:** 2024-09-28

**Authors:** Anže Feguš, Tea Sara Sagaj, Nina Fokter Dovnik, Maja Pakiž, Andraž Dovnik

**Affiliations:** 1 Medical Faculty, University of Maribor, Taborska 8, 2000 Maribor, Slovenia; anze.fegus@gmail.com (A.F.); teasarasagaj@gmail.com (T.S.S.); 2 Department of Oncology, University Medical Centre Maribor, Ljubljanska 5, 2000 Maribor, Slovenia; nina.fokterdovnik@ukc-mb.si; 3 University Clinic for Gynaecology and Obstetrics, University Medical Centre Maribor, Ljubljanska 5, 2000 Maribor, Slovenia

**Keywords:** endometrial cancer, peritoneal cytology, survival, prognostic factors

## Abstract

**Background**: Since the revision of the FIGO staging of endometrial cancer in 2009, patients with positive peritoneal cytology are no longer upstaged to stage IIIA. However, several studies demonstrated poorer outcomes in patients with positive washings. We conducted a survival analysis with the aim of evaluating the impact of positive peritoneal cytology on the survival of EC patients. **Methods**: We performed a retrospective analysis of prospectively collected data on patients with endometrial cancer operated in our institution between 2008 and 2014. We analysed the impact of positive peritoneal cytology and other established prognostic factors on survival. **Results**: A total of 227 patients with a median follow-up of 6.9 years were included in the study. A total of 12.8% had positive peritoneal cytology. Positive peritoneal cytology was significantly associated with worse overall survival (HR 1.82; 95% CI 1.02–3.26; *p* 0.043) but not with worse recurrence-free survival (HR 1.64; 95% CI 0.92–2.93; *p* 0.091) in univariate analyses. In addition, tumour stage, histologic subtype, lymphovascular space invasion, grade, and the depth of myometrial invasion were all significant prognostic factors for overall survival in univariate analyses. In multivariate survival analysis, only the histologic subtype, tumour stage, and myometrial invasion remained in the model at the last step. **Conclusions**: Positive peritoneal cytology was associated with worse overall survival in our series of endometrial cancer patients. However, other traditional prognostic factors had a more important prognostic role for survival in a multivariate model.

## 1. Introduction

Endometrial cancer (EC) is the most common gynaecological cancer in developed countries. Its incidence is rising as a consequence of the rising prevalence of risk factors such as advanced age, obesity, and metabolic syndrome [1,2,3]. The rising incidence is most prominent in high-income countries although it has also been observed in less developed parts of the world [3]. Due to the early presentation with vaginal bleeding, the majority of cases are diagnosed in early stages when the disease is confined to the uterus and the 5-year survival rates are more than 90% [4].

Prognostic factors for EC are well known. The latest European Society of Gynaecological Oncology (ESGO), European Society for Radiotherapy and Oncology (ESTRO), and European Society of Pathology (ESP) guidelines divide endometrial carcinomas into risk groups taking into consideration pathologic risk factors such as histopathological type, myometrial invasion, tumour grade, and lymphovascular space invasion [1]. Furthermore, the latest risk stratification also considers molecular classification according to The Cancer Genome Atlas (TCGA) [1].

Before the revision of the FIGO staging of endometrial cancer in 2009, positive peritoneal cytology was a stage-defining variable so that patients with positive peritoneal cytology were upstaged to stage IIIA [5]. FIGO still recommends the separate reporting of peritoneal cytology status [5]. The influence of preoperative diagnostic procedures on the intraperitoneal spread of endometrial cells has also been extensively debated [6]. Some research groups reported a higher incidence of positive peritoneal cytology after hysteroscopy compared to other diagnostic procedures, whereas others reported no such association [7,8,9,10]. A positive association between hysteroscopy and positive peritoneal cytology has also been reported in meta-analyses [11,12]. Many studies have reported an impact of positive peritoneal cytology on the survival of EC patients, and several research groups have suggested the reintroduction of positive peritoneal cytology into the staging system [13,14,15,16,17].

Following up on our previous study, we conducted a survival analysis with the aim of evaluating the impact of positive peritoneal cytology on the survival of EC patients.

## 2. Materials and Methods

### 2.1. Study Population

This retrospective analysis of prospectively collected data included the patients that were diagnosed with EC and treated with surgery in our institution between January 2008 and December 2014. Patients unfit for surgery with multiple comorbidities and patients who received neoadjuvant chemotherapy or were treated with chemoradiation were excluded from the study. All patients provided informed consent for the use of clinical data, and institutional ethical committee approval was obtained (Approval No. 13-03/15, 26 November 2015).

Before treatment, the diagnosis of EC was established with either hysteroscopy or dilatation and curettage. Tissue samples for histological diagnosis were obtained. Surgery was performed through laparoscopic or open approach. Before the start of laparoscopic surgery, a careful inspection of all visible surfaces was performed, and peritoneal washings were obtained. Irrigation of all peritoneal surfaces with saline solution was performed, and the fluid was collected from the pouch of Douglas. The sample was examined by an expert cytopathologist. After surgery, a detailed analysis of tumour histopathologic characteristics was performed, reporting on the histopathologic type, myometrial invasion, lymphovascular space invasion (LVSI), and tumour grade, together with the FIGO stage. The 2009 FIGO classification was used. Molecular classification of the tumours was not available at the time. For statistical analysis, the tumour types were divided into endometrioid and non-endometrioid cases. According to the guidelines, modified binary grading was used for this analysis with grade 1 and 2 tumours regarded as low grade and grade 3 tumours as high grade [1]. Myometrial invasion was reported as none or less than half of the myometrium vs. half or more of the myometrium. At the time, positive LVSI included focal and substantial LVSI, and negative LVSI meant absent LVSI.

### 2.2. Oncological Outcomes

Patient follow-up was conducted at our institution according to the international recommendations. Recurrence-free survival (RFS) was defined as the time from initial diagnosis to the first disease recurrence or death from any cause. Overall survival (OS) was defined as the time from initial diagnosis to death from any cause. For patients in whom no event occurred by the time of analysis (censored patients), we recorded the date of the last follow-up. The analysis included locoregional, abdominal, and distant disease relapses. The diagnosis of relapse was made clinically or radiologically, and the decision regarding the treatment of relapse was taken at the multidisciplinary team meeting.

### 2.3. Statistical Analysis

Statistical analysis was performed using the SPSS software package v. 29 (IBM, Armonk, NY, USA). Descriptive statistics, *t*-test of independent samples, and chi-square test were performed as applicable. The Kaplan–Meier method was used to calculate survival curves, and univariate Cox regression was used to assess the differences between the curves in univariate analysis. Multivariate analyses were performed by applying the multivariate Cox proportional hazards model. All variables were initially included in the Cox model, and the method used for the model was backward stepwise likelihood ratio (LR). All tests were performed at a significance level of *p* = 0.05 and a confidence interval (CI) of 95%. All *p* values were two-sided.

## 3. Results

A total of 227 patients were included in the study. Patient characteristics are presented in Table 1. More than 50% of patients were FIGO 2009 stage IA, and more than 90% of cases were of endometrioid histology.

Within the whole study population, peritoneal cytology was positive in 29 patients (12.8%). Among the 159 patients with stage I disease, 17 had positive peritoneal cytology (10.7%). The histologic characteristics of the tumours according to peritoneal cytology are presented in Table 2. There were no significant differences in peritoneal cytology status according to tumour grade and histological subtype. Positive peritoneal cytology was more common in tumours with higher disease stage, deep myometrial invasion, and present LVSI.

**Figure 1:**
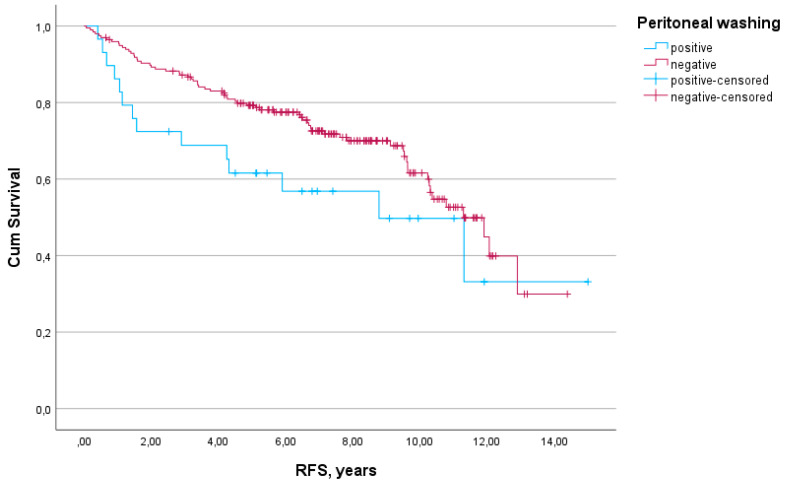
Effect of peritoneal cytology on recurrence-free survival (RFS) in endometrial cancer patients (*p* = 0.091).

**Figure 2:**
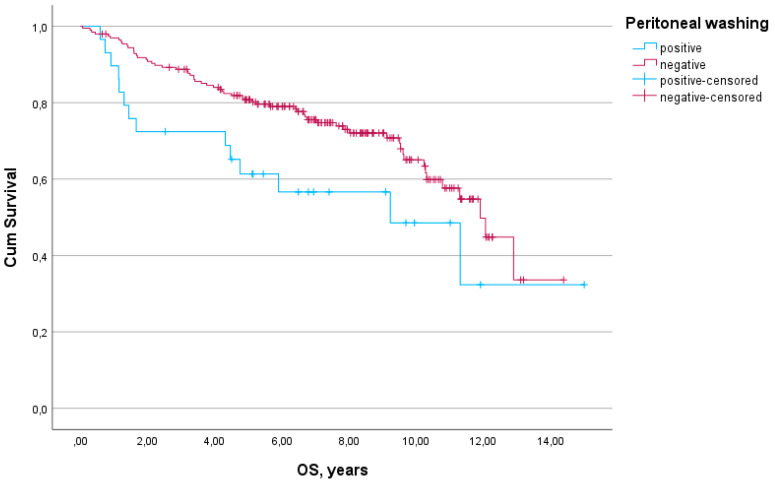
Effect of peritoneal cytology on overall survival (OS) in endometrial cancer patients (*p* = 0.043).

The median follow-up was 6.9 years. A total of 82 events occurred in the RFS analysis, and 76 events occurred in the OS analysis. The impact of positive peritoneal cytology on RFS and OS in the study population is presented in Figure 1 and Figure 2. A nonsignificant trend towards worse RFS was observed in patients with positive washings (HR 1.64; 95% CI 0.92–2.93; *p* = 0.091). OS was significantly worse in patients with positive peritoneal cytology (HR 1.82; 95% CI 1.02–3.26; *p* = 0.043).

A separate analysis for FIGO stage I patients did not confirm an impact of peritoneal washings on OS in these patients (HR 1.12; 95% CI 0.44–2.85; *p* = 0.807).

Univariate and multivariate OS analyses of different prognostic factors are shown in Table 3. In addition to peritoneal cytology, histologic subtype, FIGO stage, myometrial invasion, tumour grade, and LVSI were all found to have a significant impact on OS in univariate analyses. In backward stepwise LR multivariate model, the remaining variables significantly associated with OS after the last step were histologic subtype, tumour stage, and myometrial invasion.

## 4. Discussion

In resectable EC, positive peritoneal washings are not a very frequent finding. In our series, positive peritoneal cytology was present in 12.8% of cases, which is in line with the current literature showing positivity rates in the range of 5–23% [2,18,19]. In our study, positive peritoneal cytology correlated with deep myometrial invasion, but, contrary to some other published data, it did not correlate with tumour grade and histological subtype [2,20]. Higher rates of positive peritoneal cytology have been reported in tumours with non-endometrioid histology, positive LVSI, and more than 50% of myometrial depth invasion [4]. Matsuo et al. reported on rates of positive peritoneal cytology in patients with serous or clear-cell EC to be 10.3%, whereas the rates in low-risk endometrioid EC were as low as 3.5%. While the depth of myometrial invasion had no impact on the rates of positive peritoneal cytology in non-endometrioid EC, this finding was significantly more common in endometrioid EC with deep, compared to superficial or no myometrial, invasion [14,19].

The presence of malignant cells in peritoneal washings is suggestive of the extra-uterine dissemination of disease, which may occur spontaneously or after intrauterine procedures [21]. The spread of malignant cells through fallopian tubes appears important. A Chinese retrospective study on 562 patients with EC compared the incidence of positive peritoneal cytology in patients with and without prior tubal ligation [21]. Prior tubal ligation was associated with lower rates of positive peritoneal cytology, although both groups had similar survival rates. A subgroup analysis revealed better survival in patients with prior tubal ligation with non-endometrioid EC, but this was not the case in the endometrioid EC patients [21]. On the other hand, the results from the GOG 210 study on 4489 EC patients showed that tubal ligation was associated with better cancer-specific survival [22].

Several studies aimed to characterise the potential role of diagnostic hysteroscopy in the iatrogenic spread of malignant cells into the peritoneal cavity, yielding conflicting results [23,24,25]. A meta-analysis of 19 studies concluded that although positive peritoneal cytology occurred more commonly in patients who underwent preoperative diagnostic hysteroscopy, this did not translate into a worse prognosis for these patients [11]. Our previously published results indicated no difference in the rates of positive peritoneal cytology in patients with preoperative hysteroscopy compared to dilatation and curettage [6]. The role of uterine manipulators, which are used during laparoscopic hysterectomy in peritoneal malignant cell dissemination, is controversial, and the results from various studies are not conclusive [4].

In univariate analyses, positive peritoneal cytology, deep myometrial invasion, tumour grade, LVSI, histological subtype, and tumour stage all had significant associations with the overall survival of EC patients in our study. However, only histologic subtype, tumour stage, and myometrial invasion were retained as the most important prognostic parameters in the multivariate survival analysis.

Since the 2009 revision of the endometrial cancer FIGO classification, positive peritoneal cytology has no longer been considered as a stage-defining variable, meaning that the patients with stage I EC and positive peritoneal cytology have no longer been upstaged to stage III [5]. This modification was made as a result of studies that failed to prove the role of positive peritoneal cytology in the survival of EC patients [4]. Since this revision, many research groups have evaluated the role of positive peritoneal cytology in early stages of EC. In a single-centre study on 478 patients with stage IA low-grade endometrioid EC, Fujiwara et al. concluded that patients with positive peritoneal cytology had worse overall and 5-year disease-free survival [26]. Another Japanese multicentre analysis on 1616 EC patients showed that positive peritoneal cytology was an adverse prognostic factor in all disease stages [16]. When the patients were divided according to risk groups, positive peritoneal cytology was an adverse prognostic factor in the high- and intermediate-risk groups but not in the low-risk group [16], which is contrary to the results published by Fujiwara et al. [26]. In addition, a Canadian retrospective study on 849 EC patients showed no prognostic impact of positive peritoneal cytology in low-risk EC [27]. Furthermore, a recent Chinese retrospective study included 6313 EC patients who were stratified according to the ESGO risk groups [1,28]. Positive peritoneal cytology had no effect on survival in the low-risk group but affected the survival in intermediate- and high-intermediate risk groups [28]. In our study, tumour stage (III/IV vs. I/II) according to the 2009 FIGO classification had a more important prognostic role for survival in the multivariate analysis than positive peritoneal cytology. This finding seems to support the current system where there is no need for upstaging due to positive peritoneal cytology. In addition, a subgroup analysis showed no impact of peritoneal cytology on the survival of stage I patients in our series.

On the other hand, an American retrospective analysis of 24,800 patients with endometrioid EC within the SEER database revealed decreased cause-specific and overall survival in patients with stage I patients with positive peritoneal cytology [14]. In the high-intermediate risk group defined as per the PORTEC criteria, an improvement in the overall survival of patients with positive peritoneal cytology was seen with adjuvant chemotherapy compared to whole-pelvic irradiation [14,29]. Another analysis in endometrioid EC patients in stages I and II concluded that positive peritoneal cytology did not impact the risk of local recurrence but was associated with the increased risk of distant recurrence [17]. In an analysis of 4506 stage I non-endometrioid EC patients, positive peritoneal cytology was associated with worse survival. The 5-year survival rate was nearly 20% lower in patients with T1a disease with positive compared to negative peritoneal cytology. The authors concluded that positive peritoneal cytology is likely an independent prognostic factor unrelated to lymph node status because the results were similar in groups with and without lymphadenectomy [19].

An important consideration when discussing the role of positive peritoneal cytology in early-stage EC patients is whether this finding warrants adjuvant therapy after surgery [4]. The latest ESGO risk stratification does not include peritoneal cytology as a defining parameter when deciding the indication for adjuvant therapy [1]. However, the National Comprehensive Cancer Network (NCCN) guidelines provide a stratification of adjuvant treatment for early-stage high-risk patients depending on the presence of malignant cells in peritoneal washings [4]. For early-stage EC with clear cell or serous histology and positive peritoneal washings, the guidelines recommend a combination of adjuvant systemic treatment and vaginal brachytherapy, whereas only observation or vaginal brachytherapy is recommended in cases without malignant cells in peritoneal washings. No stratification according to peritoneal cytology is recommended for endometrioid EC [4].

The role of positive peritoneal cytology could have the greatest importance in stage I EC where adjuvant treatment is often not indicated considering other prognostic factors [4]. A retrospective analysis of the National Cancer Database including 16,851 women with stage IA-II EC reported decreased survival in patients with positive peritoneal cytology, including those with low-grade EC. The use of adjuvant chemotherapy in these women was associated with increased survival [30]. However, separate analyses of low-risk endometrioid EC patients do not seem to show a survival benefit of adjuvant chemotherapy in cases with positive peritoneal cytology [4].

Our study has several limitations. It is a retrospective single-centre analysis. At the time of the initial treatment of the included patients, molecular analyses were not yet routinely performed, and LVSI was only reported as positive or negative but not yet as substantial or focal. In addition, the absolute number of patients with positive peritoneal washings and, in some other subgroups, was rather small. In order to at least partially overcome this issue and give the reader a better understanding of the results, the findings are presented with 95% confidence intervals.

## 5. Conclusions

The results of our study indicate some prognostic value of positive peritoneal cytology in resectable EC. However, other prognostic factors, especially tumour stage, histological subtype, and the depth of myometrial invasion, were more important in the multivariate survival analysis.

## Figures and Tables

**Table 1:** Patient characteristics and histological parameters in the study population.

Study Population (N = 227)	N (%)
**Age (mean ± SD)**	66.4 ± 11.0
**FIGO stage (2009 classification)**	
IA	121 (53.3)
IB	55 (24.2)
II	16 (7.0)
IIIA	8 (3.5)
IIIB	5 (2.2)
IIIC1	12 (5.3)
IIIC2	0
IVA	2 (0.9)
IVB	7 (3.1)
**Tumour grade**	
Low grade (G1, G2)	194 (85.8)
High grade (G3)	32 (14.2)
**Histological subtype**	
Endometrioid	211 (93.0)
Non-endometrioid	16 (7.0)
**Myometrial invasion**	
Less than 50%	141 (62.4)
50% or more	85 (37.6)
**Lymphovascular space invasion**	
Present	34 (15.0)
Absent	193 (85.0)
**Surgical approach**	
Laparotomy	153 (67.7)
Laparoscopy	73 (32.3)

SD—standard deviation.

**Table 2:** Histologic characteristics according to peritoneal cytology.

	PPC [N (%)]	NPC [N (%)]	*p* Value
**FIGO stage (2009 classification)**			**0.002**
Stage I and II	19 (9.9)	173 (90.1)	
Stage III and IV	10 (28.6)	25 (71.4)	
**Tumour grade**			0.610 *
Low grade (G1, G2)	24 (12.4)	170 (87.6)	
High grade (G3)	5 (15.6)	27 (84.4)	
**Histological subtype**			0.973 *
Endometrioid	27 (12.8)	184 (87.2)	
Non-endometrioid	2 (12.5)	14 (87.5)	
**Myometrial invasion**			0.012 *
Less than 50%	12 (8.5)	129 (91.5)	
50% or more	17 (20.0)	68 (80.0)	
**Lymphovascular space invasion**			0.009 *
Absent	20 (10.4)	173 (89.6)	
Present	9 (26.5)	25 (73.5)	

PPC—positive peritoneal cytology; NPC—negative peritoneal cytology. * chi-square test.

**Table 3:** Univariate and multivariate analysis of the impact of different prognostic factors on overall survival in endometrial cancer patients (N = 227).

	Univariate Analysis		Multivariate Analysis with All Variables		Multivariate Analysis—Backward LR Model	
	Hazard Ratio (95% CI)	*p*	Hazard Ratio (95% CI)	*p*	Hazard Ratio (95% CI)	*p*
**Peritoneal cytology** (positive vs. negative)	1.82 (1.02–3.26)	**0.043**	1.34 (0.71–2.55)	0.366	-	-
**Myometrial invasion** (≥ 50% vs. < 50%)	2.06 (1.31–3.24)	**0.002**	1.80 (1.09–2.98)	**0.021**	1.99 (1.23–3.22)	**0.005**
**Histological subtype** (non-endometrioid vs. endometrioid)	4.45 (2.44–8.13)	**<0.001**	3.81 (1.80–8.06)	**<0.001**	4.49 (2.35–8.60)	**<0.001**
**LVSI** (present vs. absent)	2.75 (1.65–4.58)	**<0.001**	1.08 (0.55–2.12)	0.831	-	-
**Grade** (G3 vs. G1–2)	2.78 (1.62–4.74)	**<0.001**	1.37 (0.72–2.63)	0.340	-	-
Tumour stage * (III–IV vs. I–II)	3.55 (2.16–5.85)	**<0.001**	2.30 (1.24–4.29)	**0.008**	2.68 (1.58–4.53)	**<0.001**

LVSI—lymphovascular space invasion; * FIGO 2009 staging; The bold means statistically significant differences.

## Data Availability

The raw data supporting the conclusions of this article will be made available by the authors on request.
